# Supplementary material for: Overexpression of neuregulin 1 in GABAergic interneurons results in reversible cortical disinhibition
Source: Nat Commun. 2021 Jan 12;12:278. doi: 10.1038/s41467-020-20552-y (PMC7804852; doi:10.1038/s41467-020-20552-y)

Supplementary information

Supplementary Table 1 The demographics and the brain sample information of the subjects from database GSE 93577 and 93987
The parameters (age, PMI, pH, RIN and storage time) of the postmortem PFC samples are similar between control and schizophrenia patients. Values are means (SEM). PMI, postmortem interval (hours); RIN, RNA integrity number; Storage time (months) at -80°C; ASCVD, arteriosclerotic cardiovascular disease; ATOD, at time of death. The following probes were used: type I Nrg1 (11745036_at); type IV Nrg1 (11755968_a_at); Nrg 3 (11740174_a_at); Nrg1 in PN (208231_PM_at) and Gapdh (AFFX-HUMGAPDH/M33197_5_at). * The p value was obtained through two-sided t-test, n = 36 for each group. Data are presented as mean values (SEM).

Supplementary Table 2 Similar electrophysiological characteristics of pyramidal neurons from layer 2-3 of PrL between two genotypes * The p value was obtained through two-sided t-test, n = 27 cells from 5 control mice, n = 25 cells from 4 gtoNrg1 mice. Data are presented as mean values +/- SEM.

Supplementary Fig. 1 Nrg gene expression in GABAergic interneuron
a Similar type I *Nrg1* expression in GABAergic interneurons from postmortem PFC between drug-naïve and drug-treated schizophrenia patients. NS, not significant, two-sided t-test, n = 5 and 31 for drug-naïve and drug-treated schizophrenia patients, respectively. Data are presented as mean values +/- SEM. **b** Similar type I *Nrg1* expression in GABAergic interneurons from postmortem PFC between male and female schizophrenia patients. NS, not significant, two-sided t-test, n = 27 and 9 for male and female schizophrenia patients, respectively. Data are presented as mean values +/- SEM. **c** No significant alteration of type IV *Nrg1* expression in GABAergic interneurons from schizophrenia PFC. NS, not significant, two-sided t-test, n = 36 for each group. Data are presented as mean values +/- SEM. **d** Reduced *Nrg3* mRNA levels in GABAergic interneurons from postmortem PFC of schizophrenia patients compared with age and sex matched controls. * P = 0.0456, two-sided t-test, n = 36 for each group. Data are presented as mean values +/- SEM. Cont, control; SZ, schizophrenia; IN, GABAergic interneurons; PN, pyramidal neurons. The levels of *Nrg1* and *Nrg3* mRNA were normalized to that of Gapdh. **e** Expression of HA specifically in GABAergic interneurons from gto*Nrg1* PFC. The cellular extract from GABAergic interneurons (IN) and excitatory pyramidal neurons (PN) were subjected to single-cell RT-PCR for HA, Gapdh, vGat (IN maker) and vGlut1 (PN marker). The aCSF and tissue homogenate were used as negative and positive controls, respectively. Three independent experiments were repeated to get the similar results.

Supplementary Fig. 2 GABAergic interneuron-specific tet-off system
a The principle to generate gtoGfp mice. The *Gad67*-tTA knockin mice were crossed with TRE-H2B-GFP mice to get *Gad67*-tTA; TRE-H2B-GFP mice (i.e., gtoGfp mice). **b** The genotype and GFP expression in different mouse lines. **c** Expression of

H2B-GFP in GABAergic interneurons. The PFC slices from gtoGfp mice were immunostained with anti-GABA, the marker of GABAergic interneurons. Scale bar, 50 µm. Three independent experiments were repeated to get the similar results. **d** No expression of H2B-GFP in excitatory pyramidal neurons. The PFC slices from gtoGfp mice were immunostained with anti-neurogranin, the marker of excitatory neurons. Scale bar, 50 µm. Three independent experiments were repeated to get the similar results. H2B, histone-2B.

Supplementary Fig. 3 Body weight and behavioral assay

a Similar body weight between control and gto*Nrg1* mice at 3-month-old. NS, not significant, two-sided t-test, n = 14 for each group. Data are presented as mean values +/- SEM. **b** Similar time staying in the margin and center region of open field between control and gto*Nrg1* mice. NS, not significant, Genotype F (1, 26) = 1, P = 0.33, two-way-ANOVA, n = 14 for each group. Data are presented as mean values +/- SEM. **c-e** Similar response to prepulse noise of 75 dB (c), 80 dB (d) and 85 dB (e). NS, not significant, two-sided t-test, n = 16 for each group. Data are presented as mean values +/- SEM. **f-g** Prepulse itself has significant effects on PPI in both control (f) and gto*Nrg1* mice (g). *** P < 0.001, one-way-ANOVA, n = 16 for each group. Data are presented as mean values +/- SEM. **h** Occupancy plot of the heads from control and gto*Nrg1* mice in the three-chamber test. O, object; Mid, middle chamber; S1, social mouse. **i** Similar social interaction between control and gto*Nrg1* mice. Time spent in each chamber was quantified. NS, not significant, two-sided t-test, n = 16 for each group. Data are presented as mean values +/- SEM. **j** Better olfaction in gto*Nrg1* mice compared with controls. The buried food finding time was quantified. * P = 0.0168, two-sided t-test, n = 18 for each group. Data are presented as mean values +/- SEM.

Supplementary Fig. 4 Normal GABA release in the PFC of gto*Nrg1* mice

a Representative mIPSC traces from layer 2-3 PN in the PrL. **b-c** Similar mIPSC frequency (b) and amplitude (c) between control and gto*Nrg1* mice. NS, not significant, two-sided t-test. n = 28 cells from 4 control mice, n = 23 cells from 5 gto*Nrg1* mice. Data are presented as mean values +/- SEM. **d** Representative eIPSC traces induced by paired stimuli at an interval of 100 ms. **e** Similar PPR of eIPSC between control and gto*Nrg1* mice. Genotype F (1, 44) = 0.9897, P = 0.3253, two-way-ANOVA, n = 25 cells from 5 control mice, n = 21 cells from 4 gto*Nrg1* mice. Data are presented as mean values +/- SEM.

Supplementary Fig. 5 Normal cortical lamination and neuronal density in the PFC of gto*Nrg1*; Gfp mice

a-b Immunostaining of NeuN and GFP expression in PFC slices from gtoGfp (a) and gto*Nrg1*; Gfp (b) mice. Scale bar, 100 µm. **c** Quantification of the density of NeuN-positive cells in different layers of PFC from gtoGfp and gto*Nrg1*; Gfp mice. NS, not significant, Genotype F (1, 4) = 2.64, P = 0.18, two-way-ANOVA, n = 3 mice for each group. Data are presented as mean values +/- SEM. **d** Quantification of the

density of GFP-positive cells in different layers of PFC from gtoGfp and gto*Nrg1*; Gfp mice. NS, not significant, Genotype F (1, 4) = 1.68, P = 0.26, two-way-ANOVA, n = 3 mice for each group. Data are presented as mean values +/- SEM.

Supplementary Fig. 6 Gene expression levels of *Scn1a* (a), *Scn2a1* (b), *Scn3a* (c) and *Scn8a* (d) in the seven major neuronal clusters of adult mouse frontal cortex. The original data were from the online database DropViz (https://dropviz.org). The p value and n number were provided in the source file.

Supplementary Fig. 7 Similar mRNA and protein levels of *Scn1a* between control and gto*Nrg1* PFC

a Similar *Scn1a* mRNA levels in the PFC between two genotypes. NS, not significant, two-sided t-test, n = 3 for each group. The *Scn1a* mRNA levels were normalized to that of Gapdh. Data are presented as mean values +/- SEM. **b** Similar SCN1A protein levels in the PFC between two genotypes. Left, representative immunoblots, the total lysate of PFC from control and gto*Nrg1* mice were subjected to western blot and probed with the indicated Abs, right, quantification of SCN1A/GAPDH. NS, not significant, two-sided t-test, n = 3 for each group. Data are presented as mean values +/- SEM. **c** Similar SCN1A protein levels in the membrane fraction of PFC between two genotypes. Left, representative immunoblots, the membrane fraction of PFC from control and gto*Nrg1* mice were subjected to western blot and probed with the indicated Abs, right, quantification of SCN1A/PSD95, NS, not significant, two-sided t-test, n = 4 mice for each group. Data are presented as mean values +/- SEM.

Supplementary Fig. 8 Similar protein levels and activity of ErbB4 between control and gto*Nrg1* PFC

a Representative immunoblots. The PFC homogenate from control and gto*Nrg1* mice were subjected to western blots and probed with the indicated Abs. **b** Quantification of ErbB4/GAPDH. NS, not significant, two-sided t-test, n =3 mice for each group. Data are presented as mean values +/- SEM. **c** Quantification of p-ErbB4/ErbB4. NS, not significant, two-sided t-test, n =3 mice for each group. Data are presented as mean values +/- SEM.

Supplementary Fig. 9 NRG1 EGF domain promoted GABA release but without affecting Na_v currents in GABAergic interneurons

a Increased GABA release by NRG1 EGF domain. The amplitude of eIPSC was quantified before and after treatment with 5nM NRG1 for 5 min (indicated by the bar). **b** Representative eIPSC traces before (1), 7 min (2) and 17 min (3) after NRG1 treatment. **c** No effects of NRG1 EGF domain on Na_v currents in GABAergic interneurons. Representative current traces of Na_v channels in GABAergic interneurons treated with 5nM NRG1 or BSA (control). **d** I/V curves of Na_v channels in GABAergic interneurons treated with 5nM NRG1 or BSA (control). Treatment F (1, 19) = 0.0298, P = 0.8647, two-way-ANOVA, n = 11 cells treated with BSA, n = 10 cells treated with NRG1 EGF domain. Data are presented as mean values +/- SEM.

Supplementary Fig. 10 Full length images for all gels and blots

Supplementary Table 1

	Control	Schizophrenia	P value*
Number	36	36	
Sex	27 M，9 F	27 M，9 F	
Race	30 W，6 B	24 W，12 B	
Age (years)	48.06 (2.16)	46.86 (2.07)	0.69
PMI (hours)	17.63 (1.01)	17.98 (1.47)	0.84
Brain pH	6.76 (0.04)	6.63 (0.06)	0.07
RIN	8.32 (0.09)	8.23 (0.10)	0.52
Storage time (months)	122.21 (8.29)	125.75 (8.86)	0.77

Supplementary Table 2

	Control	gto*Nrg1*	P value*
Rheobase (pA)	160.7 ± 15.74	131.2 ± 12.76	0.1600
RMP (mV)	-68.48 ± 1.64	-67.69 ± 1.57	0.7302
Rm (MΩ)	104.1 ± 5.81	124.5 ±14.16	0.1678
1st AP latency (ms)	130.7 ± 12.33	134.8 ± 10.3	0.8024
APT (mV)	-31.65 ± 1.4	-30.99 ± 1.23	0.7402
APA (mV)	92.79 ± 0.65	95.41 ± 1.60	0.1183
Half-Width (ms)	0.97 ± 0.02	0.92 ± 0.02	0.1435
AHP (mV)	-10.27 ± 0.81	-11.19 ± 1.06	0.4872

Supplementary Fig. 1

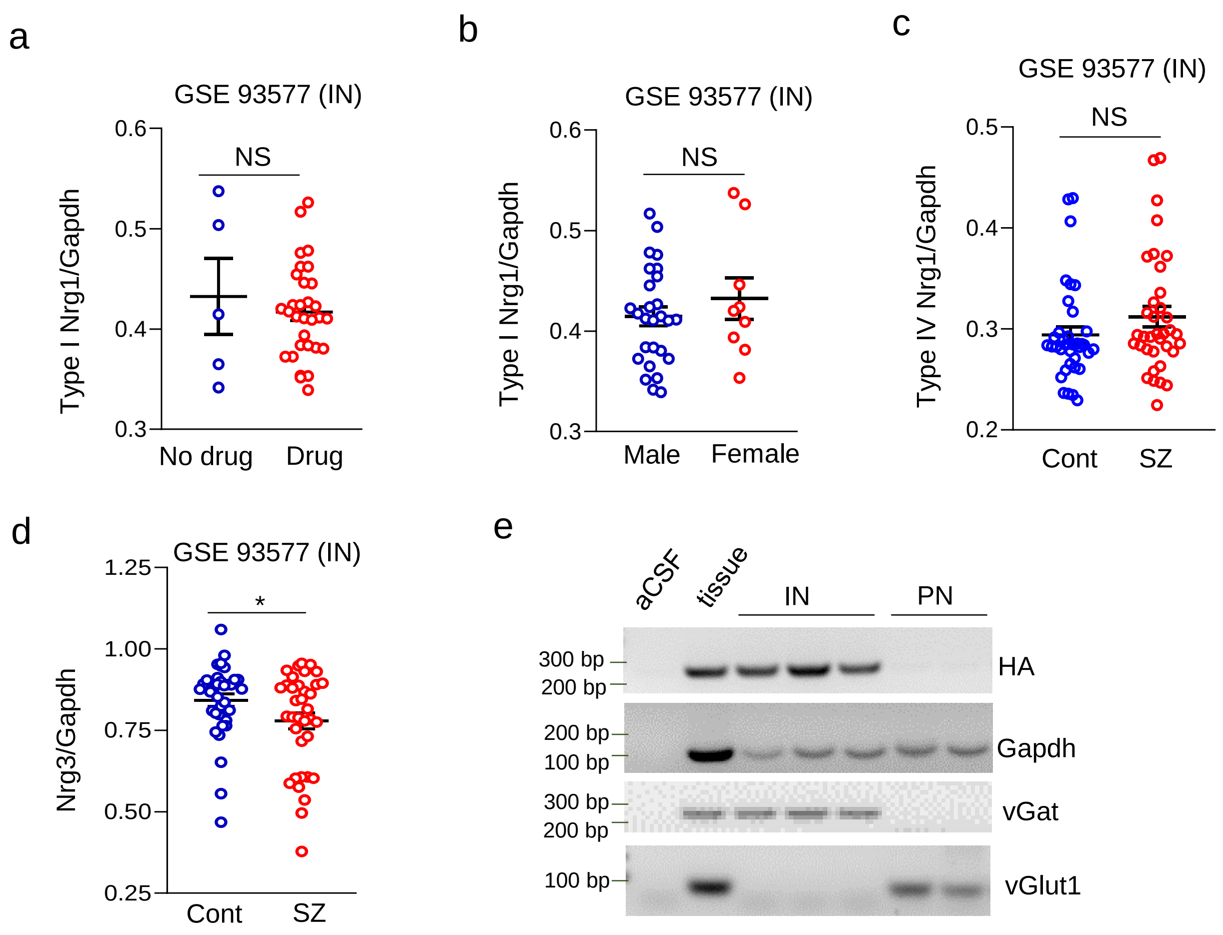

Supplementary Fig. 2

a

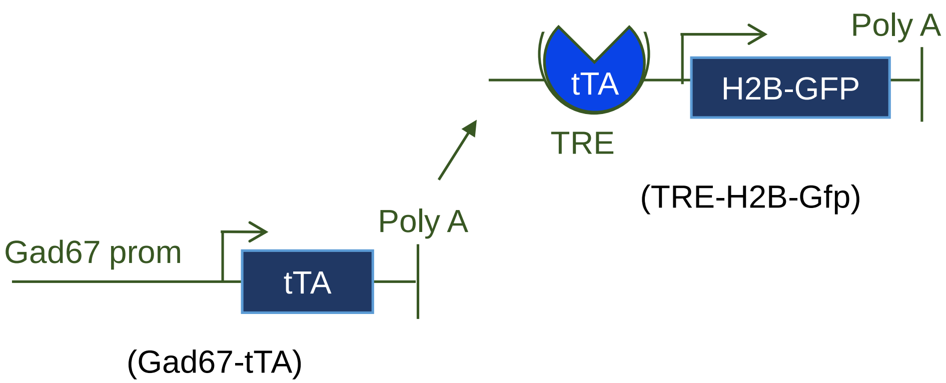

b

Genotype	GFP expression
Gad67-tTA	No
TRE-H2B-GFP	No
Gad67-tTA; TRE-H2B-GFP (gtoGfp)	Yes

c

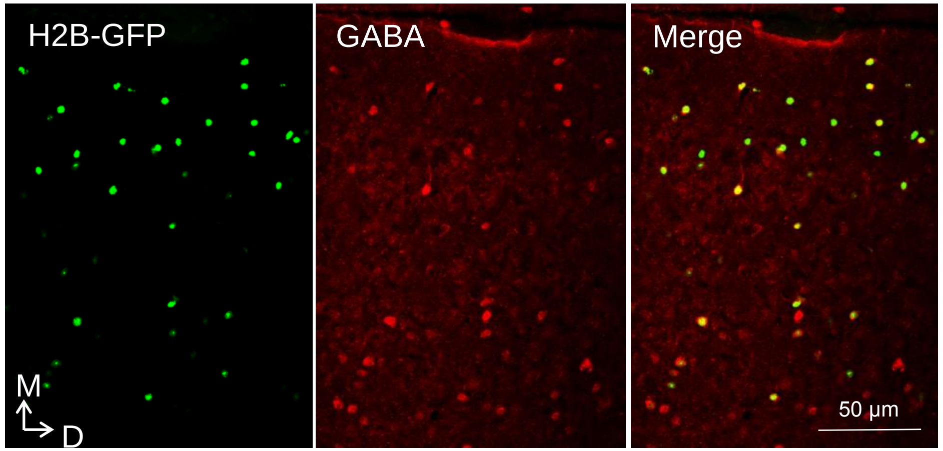

d

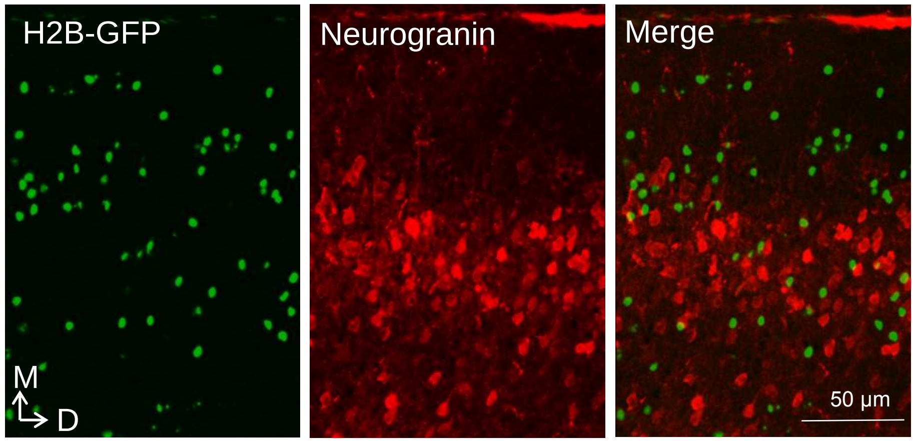

Supplementary Fig. 3

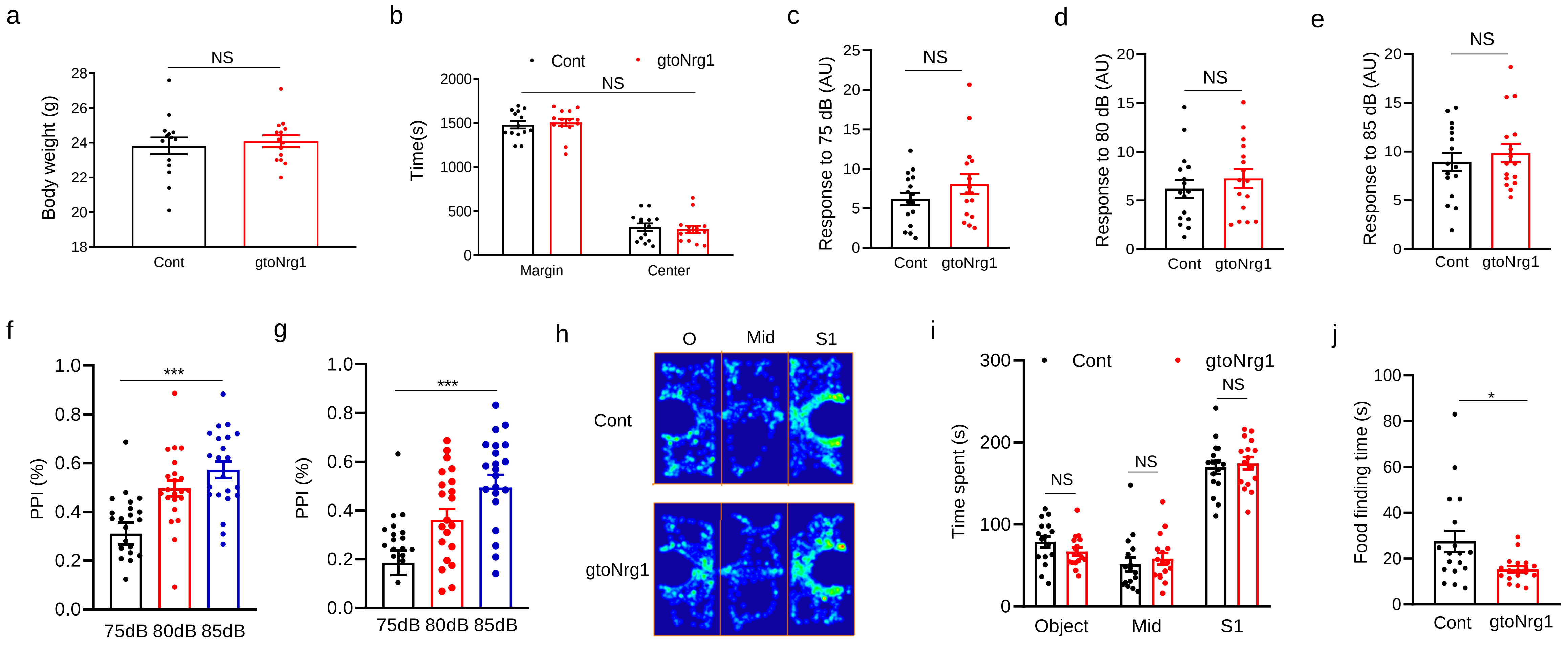

Supplementary Fig. 4

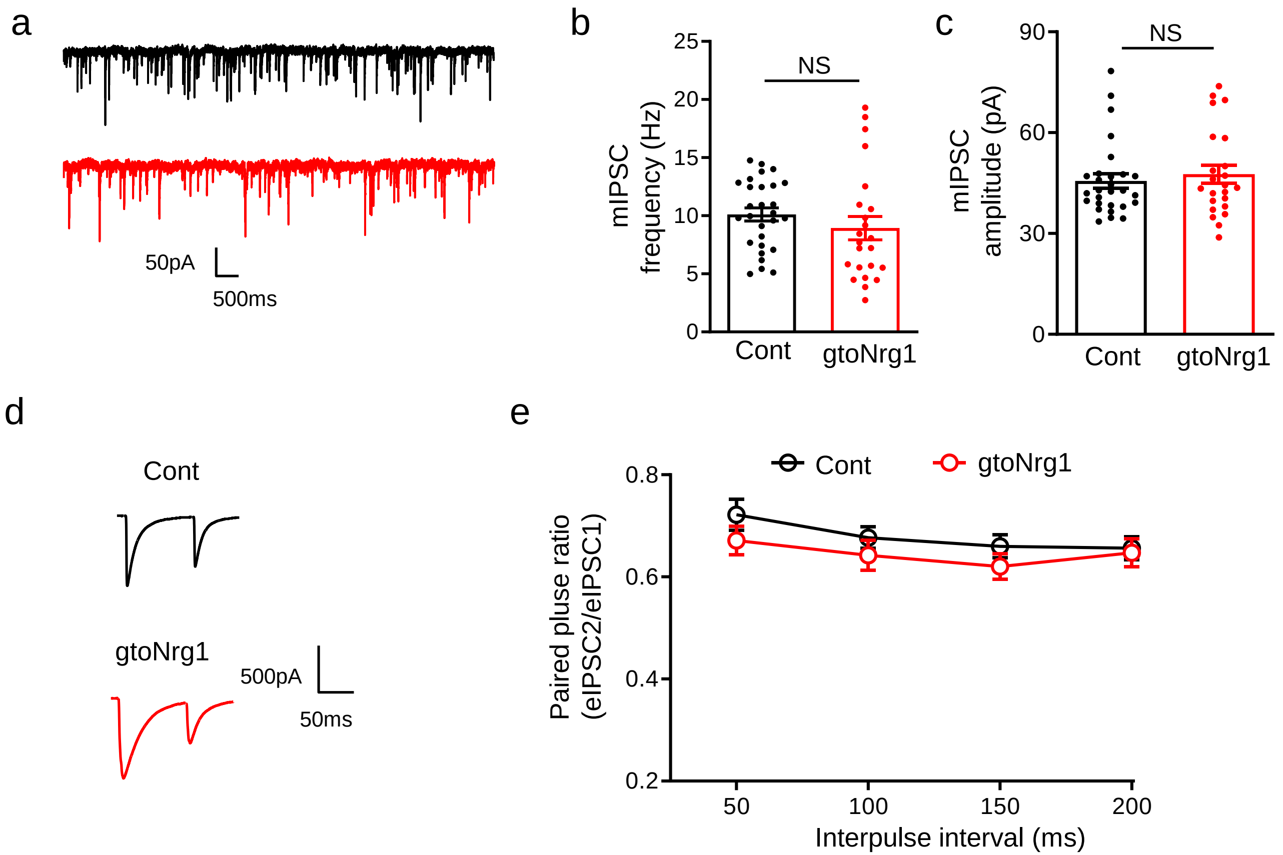

Supplementary Fig. 5

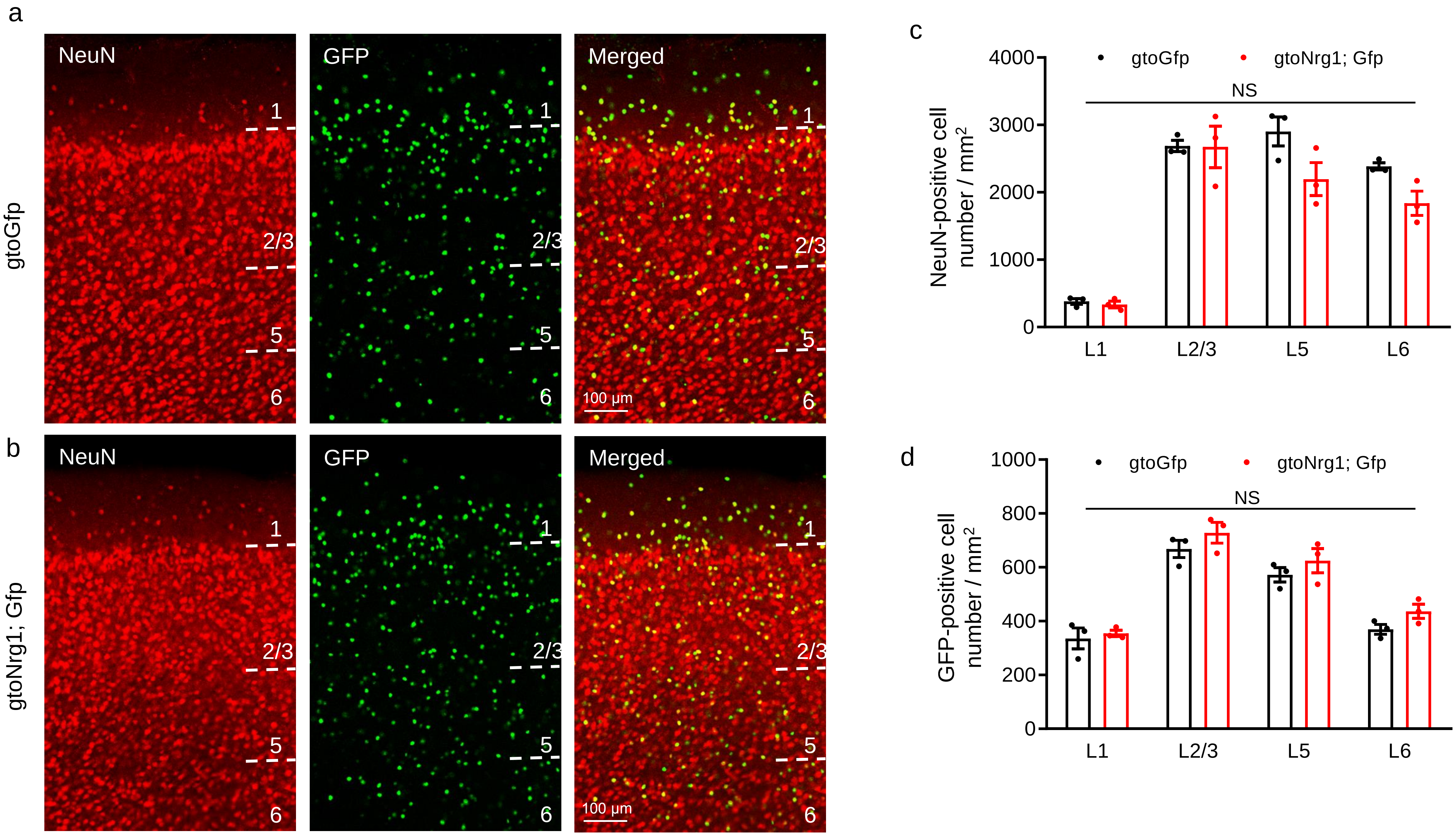

Supplementary Fig. 6

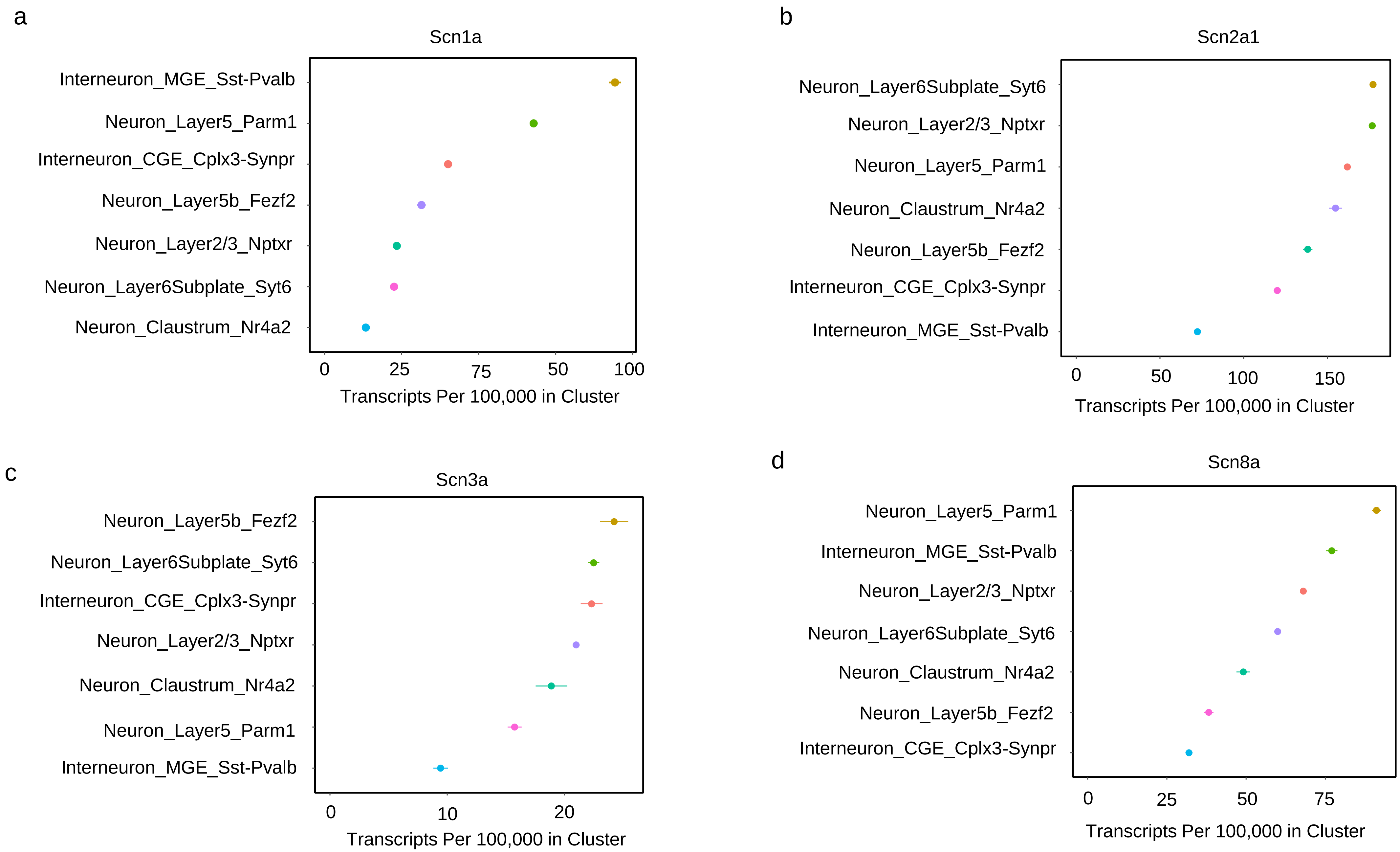

Supplementary Fig. 7

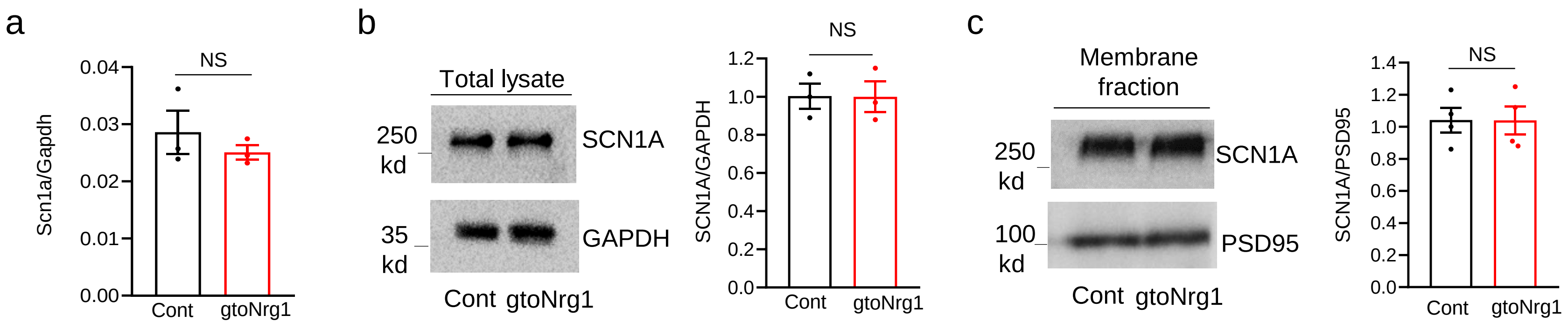

Supplementary Fig. 8

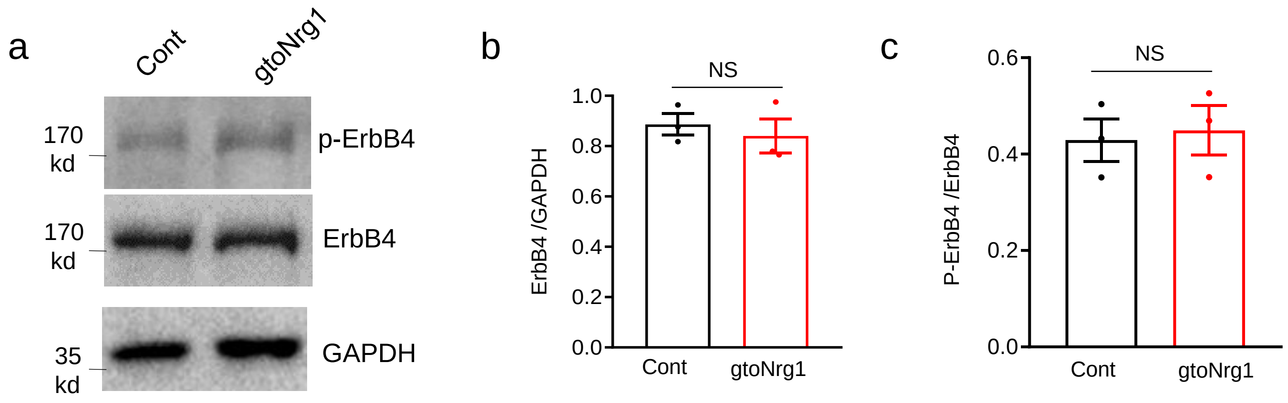

Supplementary Fig. 9

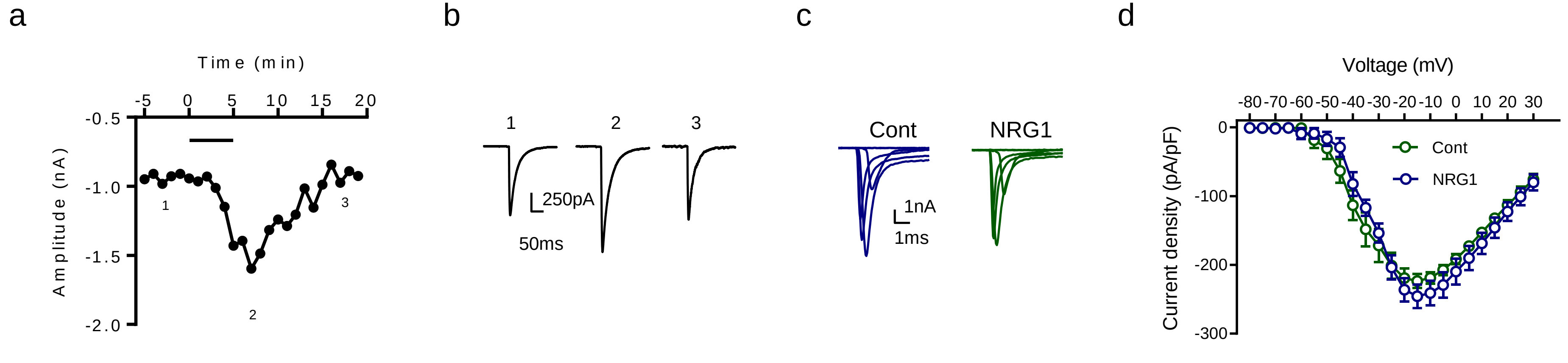

Supplementary Fig. 10

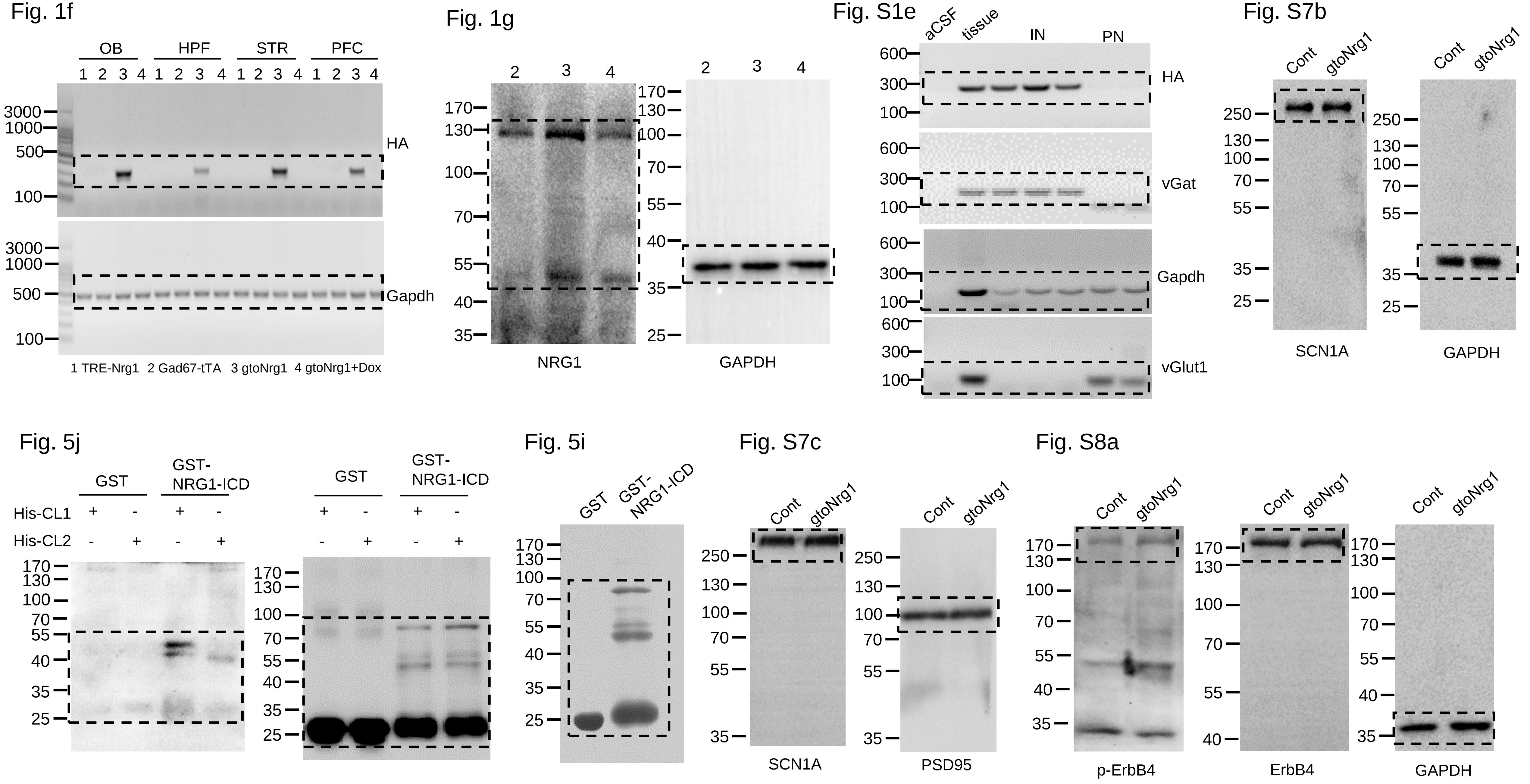

Supplement: Supplementary file 1 — Supplementary Information [file 41467_2020_20552_MOESM1_ESM.pdf]
