# Supplementary material for: Overexpression of neuregulin 1 in GABAergic interneurons results in reversible cortical disinhibition
Source: Nat Commun. 2021 Jan 12;12:278. doi: 10.1038/s41467-020-20552-y (PMC7804852; doi:10.1038/s41467-020-20552-y)
Supplement: Supplementary file 3 — Reporting Summary [file 41467_2020_20552_MOESM3_ESM.pdf]

Corresponding author(s): D Y

Last updated by author(s): Nov 26, 2020

Reporting Summary

Nature Research wishes to improve the reproducibility of the work that we publish. This form provides structure for consistency and transparency in reporting. For further information on Nature Research policies, see our Editorial Policies and the Editorial Policy Checklist.

Statistics

For all statistical analyses, confirm that the following items are present in the figure legend, table legend, main text, or Methods section.

n/a	Confirmed	
☐	☒	The exact sample size (*n*) for each experimental group/condition, given as a discrete number and unit of measurement
☐	☒	A statement on whether measurements were taken from distinct samples or whether the same sample was measured repeatedly
☐	☒	The statistical test(s) used AND whether they are one- or two-sided<br>*Only common tests should be described solely by name; describe more complex techniques in the Methods section.*
☐	☒	A description of all covariates tested
☐	☒	A description of any assumptions or corrections, such as tests of normality and adjustment for multiple comparisons
☐	☒	A full description of the statistical parameters including central tendency (e.g. means) or other basic estimates (e.g. regression coefficient) AND variation (e.g. standard deviation) or associated estimates of uncertainty (e.g. confidence intervals)
☐	☒	For null hypothesis testing, the test statistic (e.g. F, t, r) with confidence intervals, effect sizes, degrees of freedom and P value noted<br>*Give P values as exact values whenever suitable.*
☒	☐	For Bayesian analysis, information on the choice of priors and Markov chain Monte Carlo settings
☒	☐	For hierarchical and complex designs, identification of the appropriate level for tests and full reporting of outcomes
☒	☐	Estimates of effect sizes (e.g. Cohen's *d*, Pearson's *r*), indicating how they were calculated

Our web collection on statistics for biologists contains articles on many of the points above.

Software and code

Policy information about availability of computer code

Data collection	Chromo 4 (Bio-Rad), TissueFAX Plus ST (Tissue Gnostics), ChemiDocTM XRS + Imaging System (Bio-Rad), Leica TCS SP8 confocal microscope, SR-LAB TM Startle Response System (San Diego Instruments), Plexon MAP system, Vibroslice (VT 1000S; Leica), Olympus BX51WI microscope, HEKA EPC 10 double patch-clamp amplifier, PATCHMASTER version 2 x 90.1 software (HEKA), Stereotaxic apparatus (RWD Life Science).
Data analysis	Image J (NIH), Metlab R2013a, Mini Analysis Program (Synaptosoft), Igor pro 6.7.3.2 (WaveMetrics) with Neuromatic version 3.0, ANY-maze video tracking system (Stoelting), GraphPad Prism 8 (GraphPad).

For manuscripts utilizing custom algorithms or software that are central to the research but not yet described in published literature, software must be made available to editors and reviewers. We strongly encourage code deposition in a community repository (e.g. GitHub). See the Nature Research guidelines for submitting code & software for further information.

Data

Policy information about availability of data

All manuscripts must include a data availability statement. This statement should provide the following information, where applicable:

- Accession codes, unique identifiers, or web links for publicly available datasets
- A list of figures that have associated raw data
- A description of any restrictions on data availability

All data supporting the results presented herein are available from the article paper, supplementary information and source data. The full-length images for all the gels and blots are provided in Supplementary Fig. 10. Source data are provided with this paper. The web-links of database GSE93577 and GSE93987 are as follows: https://www.ncbi.nlm.nih.gov/geo/query/acc.cgi?acc=GSE93577, https://www.ncbi.nlm.nih.gov/geo/query/acc.cgi?acc=GSE93987.

Field-specific reporting

Please select the one below that is the best fit for your research. If you are not sure, read the appropriate sections before making your selection.

☒ Life sciences ☐ Behavioural & social sciences ☐ Ecological, evolutionary & environmental sciences

For a reference copy of the document with all sections, see nature.com/documents/nr-reporting-summary-flat.pdf

Life sciences study design

All studies must disclose on these points even when the disclosure is negative.

Sample size	Sample size choice was based on previous studies, not predetermined by a statistical method.
Data exclusions	According to the Wikipedia article on normal distribution, about 95% of the values lie within two standard deviations. Our approach was to remove the data that were above (Mean + 2*SD) and below (Mean - 2*SD).
Replication	Independent experiments were repeated three to four times to get the similar results.
Randomization	Randomized, some animals where not randomized due to the necessity of a genetic construct (gtoNrg1, gtoGfp mice).
Blinding	The investigators were blind to the genotype and treatment of the animals.

Reporting for specific materials, systems and methods

We require information from authors about some types of materials, experimental systems and methods used in many studies. Here, indicate whether each material, system or method listed is relevant to your study. If you are not sure if a list item applies to your research, read the appropriate section before selecting a response.

Materials & experimental systems

n/a	Involved in the study	
☐	☒	Antibodies
☒	☐	Eukaryotic cell lines
☒	☐	Palaeontology and archaeology
☐	☒	Animals and other organisms
☒	☐	Human research participants
☒	☐	Clinical data
☒	☐	Dual use research of concern

Methods

n/a	Involved in the study	
☒	☐	ChIP-seq
☒	☐	Flow cytometry
☒	☐	MRI-based neuroimaging

Antibodies

Antibodies used	rabbit anti-NeuN (1:500, Abcam, ab177487), mouse anti-GABA (1:1000, Invitrogen, PA5-32241), rabbit anti-Neurogranin (1:1000, R&D, MAB7947), rabbit anti-NRG1 (1:1000, Santa Cruz, sc-348), mouse anti-GAPDH (1:5000, Abways, ab0037), mouse anti-PSD95 (1:1000, Millipore, 2492127), rabbit anti-ErbB4 (1:1000, Cell Signaling,111B2), rabbit anti-p-ErbB4 (1:200, Cell Signaling, Y1248) and rabbit anti-Nav1.1 (1:500, Alomone Labs, ASC-001), mouse anti-GST (1:5000, ImmunoWay, B2101) and mouse anti-His (1:2000, ImmunoWay, B0401), Alexa Fluor-555 secondary antibodies (goat-anti-mouse, A32727; goat-anti-rabbit, A32732; 1:1000, Thermo Fisher) and HRP-conjugated secondary antibody (goat-anti-mouse, G-21040; goat-anti-rabbit, G-21234; 1:2000, Thermo Fisher).
Validation	The specificity of the following antibodies was validated for immunostaining in mouse by the manufacturers: anti-NeuN, anti-GABA, anti-Neurogranin. The specificity of the following antibodies was validated for western blots in mouse by the manufacturers: anti-NRG1, anti-GAPDH, anti-PSD95, anti-ErbB4, anti-p-ErbB4, anti-Nav1.1, anti-GST, anti-His. All antibodies used can be found on the corresponding websites by catalog number.

Animals and other organisms

Policy information about studies involving animals; ARRIVE guidelines recommended for reporting animal research

Laboratory animals	We used TRE-Nrg1, Gad67-tTA, TRE-H2B-GFP mice in this study. All mice are in C57BL/6N backgroud. They are 2-3 month old male mice. Animals were housed in rooms at 23°C and 50% humidity in a 12 hr light/dark cycle and with food and water available ad libitum.
Wild animals	No wild animals were used in the study.
Field-collected samples	No field collected samples were used in the study.

Ethics oversight	All experimental procedures were reviewed and approved by the Institutional Animal Care and Use Committee of East China Normal University.

Note that full information on the approval of the study protocol must also be provided in the manuscript.
